# Serial arterial lactate levels as a predictor of short- and long-term mortality in patients after cardiac surgery

**DOI:** 10.1186/2197-425X-3-S1-A104

**Published:** 2015-10-01

**Authors:** V Corral-Velez, JC Lopez-Delgado, M Rojas-Lora, N Betancur-Zambrano, N Lopez-Suñe, F Esteve Urbano, H Torrado-Santos, E Farrero, D Rodriguez Castro, L Carrio Cardona, R Mañez Mendiluce

**Affiliations:** Hospital Universitari de Bellvitge, Intensive Care, L'Hospitalet de Llobregat, Spain

## Introduction

Although hyperlactatemia is common after cardiac surgery (CS), its value and utility as a prognostic marker is controversial.

## Objectives

The aim of the present study was to determine whether postoperative serial arterial lactate (AL) measurements after CS can predict outcome.

## Methods

Prospective, observational study in our Surgical ICU in a tertiary-level university hospital. AL was measured on ICU admission,6,12 and 24h after CS, together with clinical data and outcomes including in-hospital and long-term mortality.

## Results

2935 patients were included. In-hospital and long-term mortality (mean follow-up 6.3 ± 1.7 years) were 5.9% and 8.3% respectively.In comparison with survivors, non-survivors showed higher mean AL values in all measurements(P < 0.001). Hyperlactatemia(AL>3.0 mmol/L) was a predictor for both in-hospital mortality (OR:1.468;95% CI:1.239-1.739;P < 0.001) and long-term mortality(HR:1.511;95% CI:1.251-1.825;P < 0.001).Recent myocardial infarction and longer cardiopulmonary bypass time were predictors of hyperlactatemia. The pattern of AL dynamics was similar in both groups, but non-survivors presented higher AL values, as confirmed by repeated measures analysis of variance(P < 0.001). The area under the curve also showed higher levels of AL in non-survivors(80.9 ± 68.2vs.49.71 ± 25.8 mmol·L^-1^·h^-1^;P = 0.038).Patients with hyperlactatemia were divided according to their timing of peak arterial lactate,with higher mortality and worse survival in those in whom AL peaked at 24h compared with other groups(79.1%vs.86.7-89.2%;P = 0.03).

## Conclusions

The dynamics of the postoperative AL curve in patients undergoing cardiac surgery suggests a similar mechanism of hyperlactatemia in both groups,albeit with a higher production or lower clearance of AL in non-survivors. The presence of a peak of hyperlactatemia at 24h is associated with higher in-hospital and long-term mortality in those patients.

## References

[1] Kogan A, Preisman S, Bar A (2012). The impact of hyperlactatemia on postoperative outcome after adult cardiac surgery. J Anesth.

[2] Maillet JM, Le Besnerais P, Cantoni M (2003). Frequency, risk factors, and outcome of hyperlactatemia after cardiac surgery. Chest.

[3] Ranucci M, De Toffol B, Isgrò G (2006). Hyperlactatemia during cardiopulmonary bypass: determinants and impact on postoperative outcome. Crit Care.

[4] Lindsay AJ, Xu M, Sessler DI (2013). Lactate clearance time and concentration linked to morbidity and death in cardiac surgical patients. Ann Thorac Surg.

